# Supplementary material for: Comprehensive Annotation and Functional Exploration of MicroRNAs in Lettuce
Source: Front Plant Sci. 2021 Dec 24;12:781836. doi: 10.3389/fpls.2021.781836 (PMC8739914; doi:10.3389/fpls.2021.781836)
Supplement: Supplementary file 2 [file Data_Sheet_8.ZIP › GSM2230747.plot/Lsa-miR396c_Lsat_1_v5_gn_4_159961.1_947_TPlot.pdf]

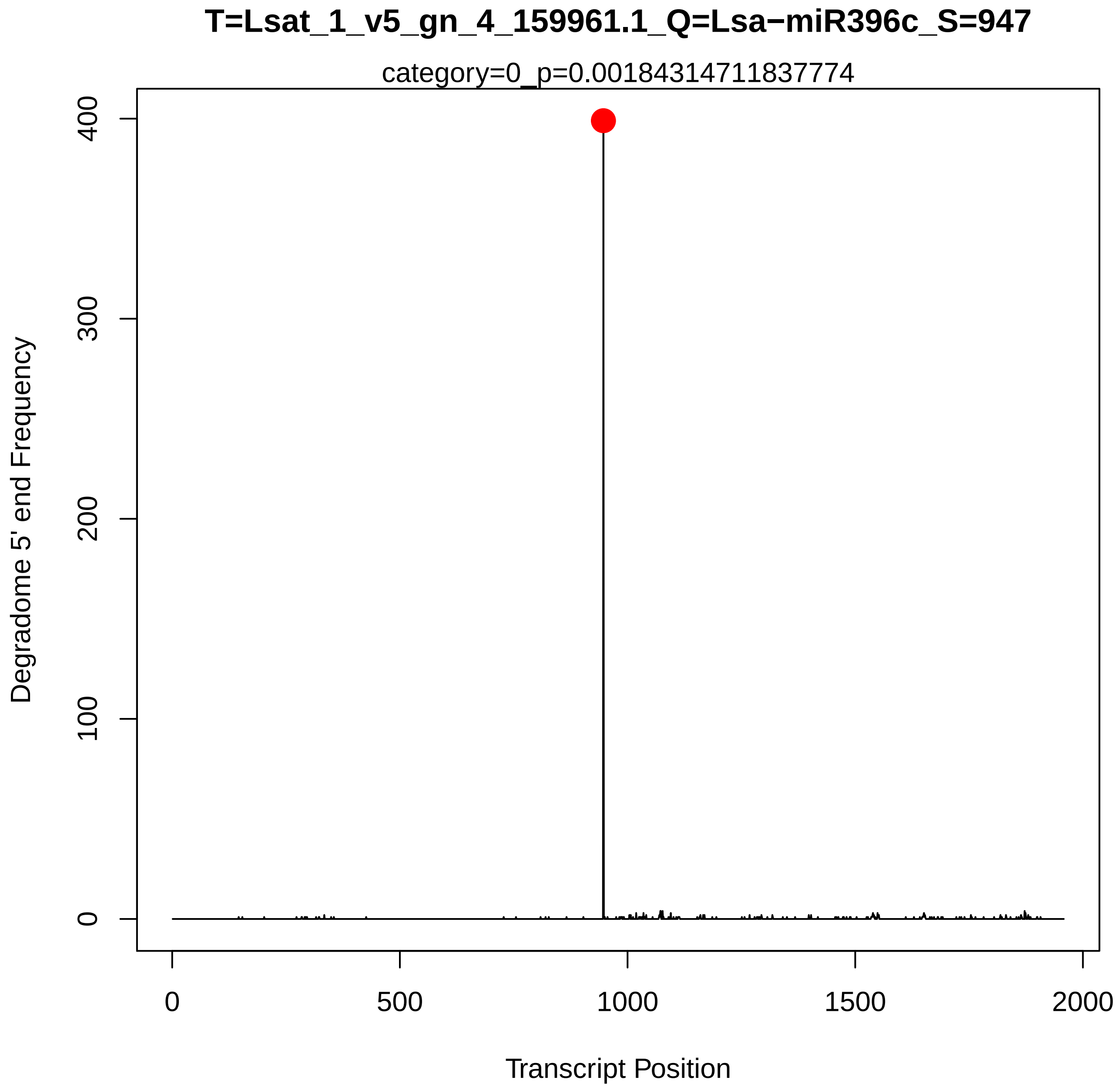

T=Lsat_1_v5_gn_4_159961.1_Q=Lsa−miR396c_S=947
category=0_p=0.00184314711837774
Degradome 5' end Frequency
400
300
200
100
0
0
500
1000
1500
2000
Transcript Position
